# Dendritic Immunotherapy Improvement for an Optimal Control Murine Model

**DOI:** 10.1155/2017/5291823

**Published:** 2017-08-20

**Authors:** J. C. Rangel-Reyes, J. C. Chimal-Eguía, E. Castillo-Montiel

**Affiliations:** Laboratorio de Simulación y Modelado, Centro de Investigación en Computación, Instituto Politécnico Nacional, Av. Juan de Dios Bátiz, Esq. Miguel Othón de Mendizábal, Col. Nueva Industrial Vallejo Delegación Gustavo A. Madero, 07738 Ciudad de México, Mexico

## Abstract

Therapeutic protocols in immunotherapy are usually proposed following the intuition and experience of the therapist. In order to deduce such protocols mathematical modeling, optimal control and simulations are used instead of the therapist's experience. Clinical efficacy of dendritic cell (DC) vaccines to cancer treatment is still unclear, since dendritic cells face several obstacles in the host environment, such as immunosuppression and poor transference to the lymph nodes reducing the vaccine effect. In view of that, we have created a mathematical murine model to measure the effects of dendritic cell injections admitting such obstacles. In addition, the model considers a therapy given by bolus injections of small duration as opposed to a continual dose. Doses timing defines the therapeutic protocols, which in turn are improved to minimize the tumor mass by an optimal control algorithm. We intend to supplement therapist's experience and intuition in the protocol's implementation. Experimental results made on mice infected with melanoma with and without therapy agree with the model. It is shown that the dendritic cells' percentage that manages to reach the lymph nodes has a crucial impact on the therapy outcome. This suggests that efforts in finding better methods to deliver DC vaccines should be pursued.

## 1. Introduction

Immunotherapy based on antigen-treated dendritic cells (DCs) is a promising treatment against certain types of cancer [1]. This kind of therapy is often regarded as a safe option alone or in concurrence with other therapies [2]. Nonetheless, clinical evidence of its efficacy is still unclear and researchers are still designing new experiments in mice and humans to decipher mechanisms that could lead to a successful cancer treatment. Murine models are a current way to test hypothetical therapy protocols by cultivating dendritic cells* in vitro* from mouse bone marrow to be inserted back into the mouse after being treated with antigens. For example, modest clinical success has been observed in C57BL/6S mice inoculated with B16 melanoma cells [3] after being treated with antigen loaded DCs.

Therapy schedules consist in previously planned protocols of injection times with their respective vaccine quantity. Such protocols in immunotherapy are usually proposed following therapist traits such as intuition and experience. We aim to provide a rational therapy planning not only relying on therapist's experience and intuition but also by the help of mathematical modeling, optimal control, and simulations. Optimal control has a history as a tool to improve tumor therapy schedules. It is usually used to rationalize issues like infusion times and drug quantities. For instance, the optimal control for a model with mixed immunotherapy and chemotherapy is addressed in [4]. Numerical and analytic control techniques for continuous and bang-bang controls can be consulted in [5]. Therapeutic protocol improvement by simulations have been made in [6] for Cytotoxic T Cell (CTL) and in [7] for dendritic cell transfections.

Maturation of antigen-primed DCs reduces phagocytic capacities in an interchange for improving and presenting migration capabilities [2] to lymph tissues increasing expression of chemokines and adhesion molecules. DCs are characterized by improving the immune response activating natural killer cells and naive and memory B cells. Moreover, DCs create cytokines which help CD4^+^T cells differentiation. Several methods to generate and mature DCs are able to produce antigen-specific T cell response after DCs inoculation. Maturation is essential since mature DCs have a greater capacity to create cytokines and chemokines that activate T cells in contrast to immature DCs.

Tumor cell antigens need to be loaded into Major Histocompatibility Complex (MHC) molecules of the DCs in a process called antigen loading [2]. For such an endeavor DCs are curated with peptides and proteins from tumor cells. Another method to load antigens to DCs is by using virus vectors. Such viruses are used to load genes with encoded Tumor Associated Antigens (TAA) into the dendritic cells. Also, the natural immune response against virus vectors serves as an immunostimulant towards the TAA.

DCs are regarded as the best at activating cytotoxic T cells. They have a history of being used as the therapy to improve the host immune capabilities. Nonetheless, many clinical results of dendritic immunotherapy are not quite successful. The purpose of this work is to propose a model that accounts for the obstacles that dendritic cells could face when they are used as a medium to transmit antigens to the host immune system and create immune response. Such obstacles are as follows:

- Only 5% of the DC vaccine reaches the lymph node [8].
- Injected DCs do not directly activate CD8 cells; they only transmit the antigens to the endogenous Antigen Presenting Cells (APCs) which are responsible for activating the T cells [9]. We suppose that DCs only interact with CD4 T cells and IL-2 cytokine.

We also include the immunosuppressive effects of the TGF-*β* in the cytotoxic T cells. Then, the model is used together with optimal control to overcome such obstacles. In this case, optimal control is used to predict satisfactory dendritic injection times with an optimal control technique developed by Castiglione and Piccoli [10]. Another consideration is regarding the kind of DCs vaccine. We do not consider a continuous infusion of DCs, but a short period or bolus injection that can be considered instantaneous. This resembles more accurately the procedure following the immunotherapy injection in [3] for which we adjusted our model.

The outline of this work is as follows. The explanation of the mathematical model and its terms are given in Section 2, followed by numerical simulations that agree with experimental data from mice bearing melanoma cells in Section 3.  Section 4 contains the optimal control problem and the optimization algorithm, followed by Section 5 that explores the consequences of changing the percentage of dendritic cells arriving at the lymph node. Finally, Section 6 gives some biological implications of the results.

## 2. The Murine Mathematical Model

The clinical efficacy of DC vaccines by simulations of mathematical models is a topic of ongoing research in mathematical oncology [1]. One of the most used tools for modeling the intricate interactions between proteins, lymphocytes, and tumor cells is ordinary differential equations (ODEs). Other kinds of models include delay differential equations (DDEs), which are a system of ODEs where an unknown depends on past values. This is usually represented in the independent variable as *t* − *τ* (e.g., *x*(*t*) and is changed for *x*(*t* − *τ*)) where *τ* is called the delay. Such delay can elegantly represent a gestation time or a transport delay, hiding some of the intricacies of the phenomena. The negative side is that it is often more difficult to solve a DDE analytically and numerically than an ODE. Several DDE models have been used to hide the transport delay of DCs from the injection time to the interaction time with effector and helper T cells. For example, a compartment model between the spleen, blood, and tumor with a delay between the DCs injection and spleen arriving is given in [11]. A DDE with interactions between TGF-*β* and T cells is made in [7].

For the model considers an inherent delay *τ* = 232 h, which is equal to the time that DCs take to arrive at lymph nodes but only affects the times at which the simulations assume an injection and is not implicitly adding *t*. Then, our model is an ODE. Also, we are considering that therapy consists of bolus injections with very small timespan in comparison with overall therapy. The details of implementation are explained in The Simulation.

### 2.1. The ODE System

The model of the present paper having been inspired by the works of [6, 7, 10] is given by the ODE system:

(1) dTdt=rTTlog⁡KTT−aTCTMIeT+MIaTFβ+eTeT+Fβ,

(2) dHdt=aH+rHDH1−HKH−μHH,

(3) dCdt=aC+rCIC1−CKC−μCC,

(4) dDdt=−μDCD+u,

(5) dIdt=−μICCI+rIDH−μII,

(6) dFβdt=rTβT−μβFβ,

(7) dFγdt=aγCC−μγFγ,

(8) dMIdt=aMlγFγeMlγ+Fγ+gMl−μMlMI

which has 8 state variables:

- *T*, the tumor cells.
- *H*, the CD4 T helper cells.
- *C*, the CD8 T or CTL cytotoxic cells.
- *D*, the antigen loaded dendritic cells.
- *I*, the IL-2 Interleukin-2 cytokine.
- *F*_*β*_, the TFG-*β* T cell inhibitor.
- *F*_*γ*_, the IFN-*γ* which upregulates MHC class 1.
- *M*_*l*_, is the number of MHC class 1 receptors per melanoma cell.

**Figure 1:**
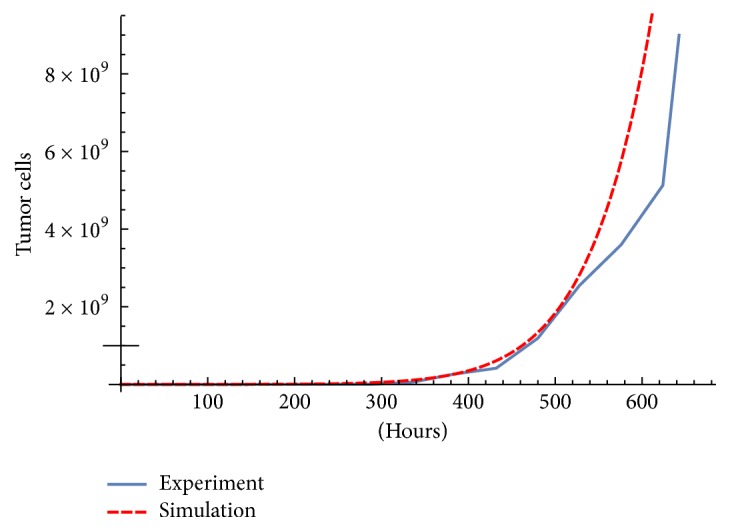
Simulation and experimental data without therapy. Parameter Table 2.

**Figure 2:**
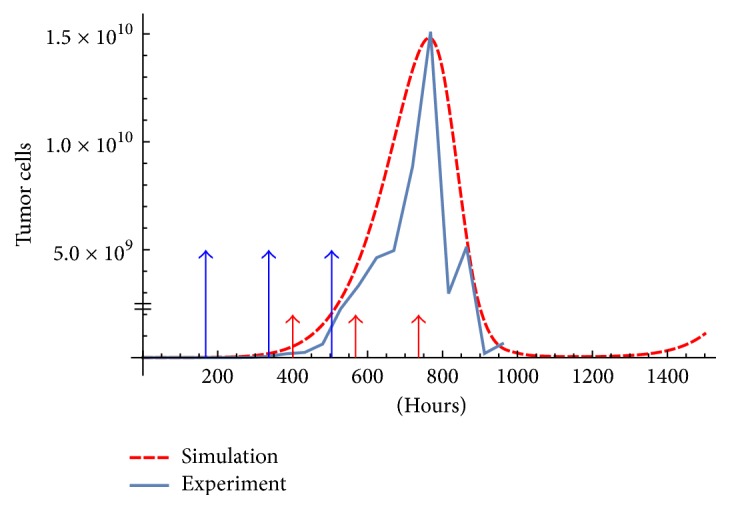
Simulation and experimental data with therapy. The experiment was obtained starting with 6 × 10^4^ melanoma cells inoculated in mice. Following a dosing protocol of 10^6^ DC at times *t* = 168,336,504 [3, 7] with total dose of 3 × 10^6^ DCs. The initial condition is 6 × 10^4^ for tumor cells and zero for the rest of the variables. The parameters used are shown in Table 2. The big arrows are the injections, and small arrows are the delayed impulses *u*.

The first term of (1) is a Gompertz growth which adjusted very well to experimental data; see Figures 1 and 2. The expression in the second term −*a*_*T*_*CT*(*M*_*I*_/(*e*_*T*_ + *M*_*I*_))((*a*_*T*_*F*_*β*_ + *e*_*T*_)/(*e*_*T*_ + *F*_*β*_)) represents the tumor cell eliminations by the CD8 T cells, which in turn is suppressed by the cytokines TFG-*β* and the efficiency of MHC class 1 receptors. Both suppressing effects follow Michaelis-Menten saturation dynamics; see [6].

Equations (2) and (3) represent the dynamics of CD4 and CD8 T cells, respectively. The parameters *a*_*H*_ and *a*_*C*_ represent the production of CD4 and CD8 T cells. Both follow a logistic growth law. As mentioned before, it is supposed that the injections of DCs only interact with CD4 T cells since it was reported in [9] that CD8 activation is not made by DC vaccines. So, in the term *r*_*C*_*IC*(1 − *C*/*K*_*C*_) we consider that activation of tumor-specific CD8 is only promoted by IL-2.

The term *u* in (4) is a control variable representing the DCs injections. Since we are considering a bolus injection therapy, *u* is the sum of impulses at injection times: *u* = *Vδ*(*t* − (*t*_*i*_ + *τ*)). Where the therapy consists of *n* doses of size *V* given at times *t*_0_ < *t*_1_ < ⋯<*t*_*n*−1_, but because there is a delay in the DCs arriving time at lymph nodes the impulse occurs at (*t*_*i*_ + *τ*).

Equation (5) have interleukin IL-2 produced from the interactions of DCs and CD4 T cells by *r*_*I*_*DH* and is consumed by CD8 T cells at −*μ*_*IC*_*CI*. Interleukin IL-2 has a major role in the system, prolonging persistence of CD8 T cells. It is necessary for the production of new tumor antigen-specific CD8 T.

Equation (6) describes the cytokine dynamics of TGF-*β*, an inhibitor of T cells activity, with secretion proportional to the number of tumor cells and degradation rate *μ*_*β*_.

Equation (7) describes the cytokine IFN-*γ*, a weapon of the CD8 T cells to upregulate the MHC class 1 in the melanoma cells. The production is proportional to the CD8 T cells and degradation rate *μ*_*γ*_.

MHC 1 class dynamics is in (8). It has a basal production rate per melanoma cell *g*_*M*_*l*__. The first term shows a growing rate stimulated by IFN-*γ* which follows a Michaelis-Menten Kinetics with maximal effect *a*_*M*_*lγ*__ and Michaelis parameter *e*_*M*_*lγ*__.

This model assumes the following:

1. The tumor mass is homogeneous. The present model assumes that each tumor cell has the same sensitivity to DCs vaccine.
2. It is monoclonal, that is, only T cells are able to recognize the TAA.

## 3. The Simulation

The model is calibrated to fit the data supplied in [7] made at the Laboratory of Immunotherapy and Tissue Engineering of UNAM (Universidad Nacional Autonoma de México), Mexico [3]. The immunotherapy consisted of three doses of 10^6^ DCs activated with MAGE-AX separated by 168 hours, in Table 1. The tumor cells population is calculated from mice tumor diameter assuming a spherical form. The used parameters can be seen in Table 2. The initial condition is (*T*(0), *H*(0), *C*(0), *D*(0), *I*(0), *F*_*β*_(0), *F*_*γ*_(0), *M*_*I*_(0)) = (6 × 10^4^, 0,0, 0,0, 0,0, 0). A comparison between the experiment and simulations with and without therapy is shown in Figures 1 and 2.

As mentioned before, in the simulation there is an inherent delay of *τ* = 232 h, which was adjusted to fit the experimental data. Hence, we implement *τ* to affect the impulse time of the control variable *u*. Therefore, for example, the therapy consists of a single injection of 10^6^ DCs at *t* = 10 h; we have

(9) $$\begin{array}{c} u=10^{6}\delta \left(\;t-\left(\;10+\tau \;\right)\;\right), \end{array}$$

where *δ* is the Dirac delta function. Observe that this implies that the DC injection at *t* = 10 h has an effect on the system until *t* = 10 + *τ*. So, we integrate our system from [0,10 + *τ*, and then restart the integration from *t* = 10 + *τ* with initial condition (*T*, *H*, *C*, *D* + 10^6^, *I*, *F*_*β*_, *F*_*γ*_, *M*_*I*_)|_*t*=10+*τ*_.

## 4. Optimal Control

Hypothetical protocols can be found simulating the model with several combinations of dose timing and size. This could lead to the implementation of new protocol therapies which could potentially replace the originally proposed by medics. For example, see [7]. Now in this section, we show that such hypothetical protocols can be derived or improved using an optimization algorithm based on optimal control.

It is considered that a therapeutic protocol consists of *n* doses of size *V* given at times *t*_0_ < *t*_1_ < ⋯<*t*_*n*−1_. Now, let a schedule of injections be given by *S* = {*t*_*i*_ : *i* = 0,1,…, *n* − 1, *t*_0_ + *τ* < *t*_1_ + *τ* < ⋯<*t*_*n*−1_ + *τ* < *t*_*f*_}, where *t*_*f*_ is the fixed time horizon. Let *ℒ* be the space of schedules, then for a particular schedule *S* ∈ *ℒ* the control variable takes the form:

(10) $$\begin{array}{c} u=\overset{n}{\underset{i=1}{\sum }}e_{f}V\delta \left(\;t-\left(\;t_{i}+\tau \;\right)\;\right), \end{array}$$

where *e*_*f*_ is the percentage of DC vaccine that actually reaches the lymph nodes. Notice, we are approximating the doses as an impulse given by the Dirac function *δ*(·). Now let *x* = (*T*, *H*, *C*, *D*, *I*, *F*_*β*_, *F*_*γ*_, *M*_*I*_), so Equations (1)–(8) take the form,

(11) $$\begin{array}{c} \frac{dx}{dt}=f\left(\;x\;\right)+u. \end{array}$$

The optimal control problem consists in the following:

(P) Determine the schedule *S* ∈ *ℒ* of *n* injections that minimize the final tumor mass *T*(*t*_*f*_) of the trajectory given by (11) with initial condition *x*_0_ = (6 × 10^4^, 0,0, 0,0, 0,0, 0).

To solve (P) we use the optimization algorithm described in [10], which is repeated here for clarity, with a slight modification.

### 4.1. Optimization Algorithm

**Algorithm 1 (optimization).**

(S0) Fix the time horizon *t*_*f*_, the number *n* of vaccine administrations, the value *V* of vaccine quantity, an initial value *x*_0_ of cells population, and an initial schedule *S*_0_.
(S1) Integrate system (1)–(8) with initial value *x*_0_ to obtain the trajectory *x*_*s*_. Solve for each *t*_*i*_ of the schedule *S*_0_:
  (12) $$\begin{array}{c} \frac{dv_{i}}{dt}=D_{x}f\left(\;x_{s}\;\right)\cdot v_{i} \end{array}$$
in the interval [*t*_*i*_, *t*_*f*_] with initial condition *v*_*i*_(*t*_*i*_) = *f*(*x*_*s*_(*t*_*i*_)) − *f*(*x*_*s*_(*t*_*i*_) + **e**_4_*V*) where **e**_4_ is the fourth coordinate vector.
(S2) Update the schedule *S*_0_ : *t*_*i*_^*n*+1^ = *t*_*i*_^*n*^ − *hv*_*i*_(*t*_*f*_) · **e**_4_ with *h* > 0. The step size *h* should be of the order *O*(1/|*v*_*i*_(*t*_*i*_) · **e**_4_|) to avoid an excessive big step. Go to step 1.

The correct choice of step size *h* is important. If *h* is too big, we could get a wrong optimization step. If *h* is too small the optimization could improve very slowly. Final tumor mass was decreased in each optimization step choosing *h* of the order *O*(1/|*v*_*i*_(*t*_*i*_) · **e**_4_|). Also, to find the best *h* an 1*D* search method could be used such as the Golden Section Search (GSS) [12]. Using GSS we achieve a good optimization with 10 steps in about 10 seconds. In contrast to a fixed step *h* it needs 100 optimization steps in 60 seconds.

We tested Algorithm 1 with an initial random schedule {232,462,692,922} and 100 optimization steps with *V* = 6 × 10^5^. Also, in order to get *x*_*s*_ at step 2 we used the same initial condition: *x*_0_ = (6 × 10^4^, 0,0, 0,0, 0,0, 0) as that used to test the model (Figure 2). This evolved into one big injection of 1.8 × 10^6^ at *t* = 303 h and one of 6 × 10^5^ at *t* = 879 h. Observe that a total dose of 2.4 × 10^6^ DCs is used, whereas the original protocol used 3 × 10^6^ DCs (Figure 2).

The difference of initial and optimized therapy is shown in Figure 5. Moreover, Figure 5(b) shows a maximum of roughly 3.5 × 10^9^ tumor cells and final tumor mass of around 5 × 10^8^; substantially less than in the experiment. The maximum and final tumor mass is 1.5 × 10^10^ and 6.41 × 10^8^ respectively, see Figure 2.

**Figure 3:**
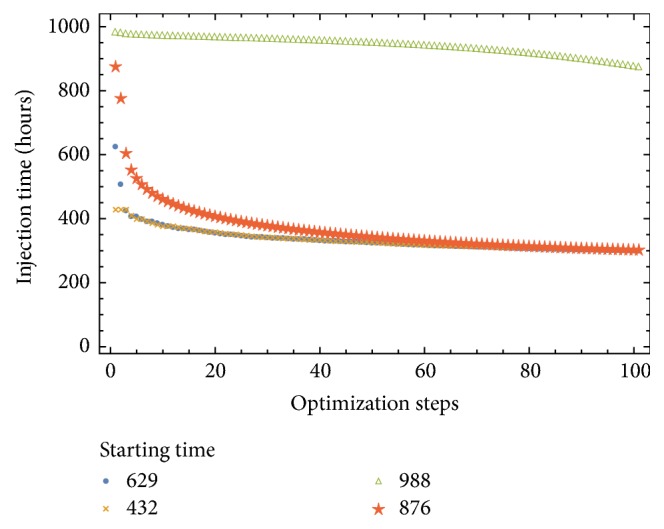
Injection schedule versus the optimization steps. With initial random protocol {432,629,876,988} and final protocol {303.636,303.634,303.211,879.318} and fixed step size *h* = 10^−6^.

**Figure 4:**
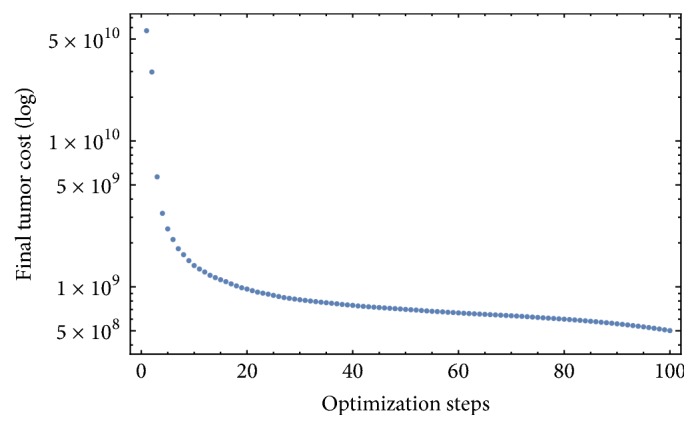
Final value of the tumor for each optimization step.

Figure 4 shows that a fast final tumor mass decay occurs in the first 10 optimization steps with little improvement afterwards. Nonetheless, Figure 3 shows a substantial therapy evolution until about the 80th optimization step.

### 4.2. Optimal Therapies in Large Time Horizons

A very slow convergence and unsatisfactory results of Algorithm 1 are observed searching for protocols far beyond *t*_*f*_ = 1000 h (e.g *t*_*f*_ = 20000 h). In view of such problems, we divide the time horizon *t*_*f*_ in *m* equal intervals of size *t*_*f*_/*m* and apply Algorithm 1 at every interval [(*i* − 1)(*t*_*f*_/*m*), *i*(*t*_*f*_/*m*)] for *i* = 1,…, *m* in an iterative way. At the end of each iteration step the state *x*(*i*(*t*_*f*_/*m*)) is saved to be used as an initial condition in the following iteration. Also, in each iteration the optimized schedule *S*_*i*_ is saved. Finally, the system is solved with the schedule *S* = ⋃_*i*=1_^*m*^*S*_*i*_.

**Algorithm 2 (optimization of the intervals).**

(S0) Fix the time horizon *t*_*f*_, number of intervals *m*, the number *n* of vaccine administrations on each interval, the value *V* of vaccine quantity, an initial value *x*_0_ of cells population and an initial schedule *S*_0_. Let *i* = 1 and *S* = *S*_0_.
(S1) If *i* equals *m*, go to Step  3. Otherwise apply Algorithm 1 at interval [(*i* − 1)(*t*_*f*_/*m*), *i*(*t*_*f*_/*m*)] with initial condition *x*_*i*−1_(*i*(*t*_*f*_/*m*)). Get the schedule *S*_*i*_.
(S2) Let *S* = *S* ∪ *S*_*i*_. Save the next initial condition *x*_*i*_(*i*(*t*_*f*_/*m*)), Let *i* = *i* + 1. Go to Step  1.
(S3) Solve the system with the schedule *S*.

## 5. Results and Discussion

The objective of this section is to show that the percentage of DC injection which arrives at lymph nodes (*e*_*f*_) has a great impact on the tumor mass behavior. Three therapy cases are shown, each starting with a random therapy which is improved by Algorithm 2.

For ethical reasons, melanoma inoculated mice are allowed to live maximum of 1000 h. Then, in order to find a therapy after such time we apply Algorithm 2 for timespans: 20,000, 10,000, and 5,000 hours; in addition, we change *e*_*f*_ from 5% to 8% and to 10%. In every case tumor oscillations with amplitude that stabilize after some time are obtained. This stabilization time seems to be smaller as *e*_*f*_ grows. It is worth mentioning that *M*_max_, the maximum number of melanoma cells that mice can bare before dying cannot be greater than 1.6 × 10^10^ [7]. So, the immunotherapy protocol with *e*_*f*_ = 0.05 shown in Figure 6 can be regarded as a successful therapy to control the tumor under *M*_max_.

When *e*_*f*_ is increased, Figures 7 and 8 show a therapy with better tumor-killing performance than therapy with *e*_*f*_ = 0.05. Using *e*_*f*_ = 0.08 (Figure 7) reduces the maximal oscillation amplitude to 3 × 10^9^ and reduces the amplitude of the stable oscillations to 7.75 × 10^8^. Finally, *e*_*f*_ = 0.10 in Figure 8 reduces the amplitude of the stable oscillations to the order 10^6^ which in some instances is regarded as clinically not detectable.

The increments of *e*_*f*_ can be implemented in experiments using a secondary therapy that improves the migration capacity of the DCs. Also, alternative delivering methods for DCs injections could be used. Nevertheless, alternative delivering routes such as intradermally and intranodally showed a comparable response to the intravenously route [8].

### 5.1. Further Research

**Figure 5:**
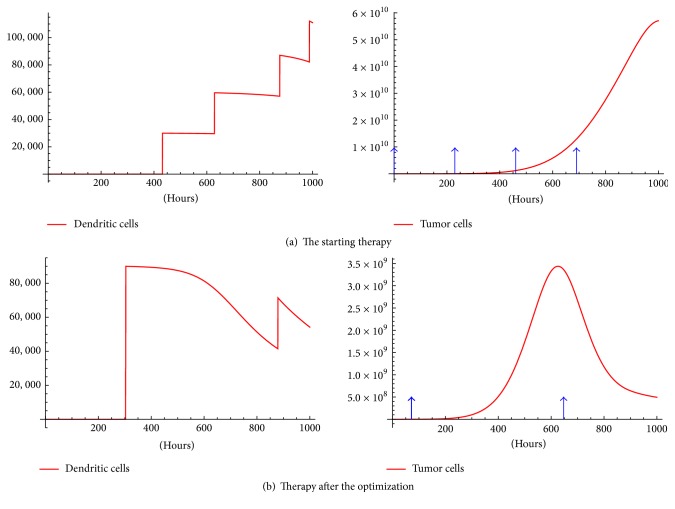
Before and after optimization procedure. The arrows represent injection times and the jumps on DCs are the impulses on the control. A total dose of 2.4 × 10^6^ DCs is used.

The overall schema followed in Algorithm 1 is called the steepest descent. This relies on following the correct gradient in each step until a minimum of the cost function is apparent. The gradient for our problem is given by (12) and only considers the cost function *T*(*t*_*f*_) [10]. Despite this, the simulations in Figure 5 show improvements over the originally used therapy. However, there are many cost functions which can be minimized. For example, a cost function that admits the total dose given over the optimization can be proposed. This could help therapists to economize on materials which cost a lot of money and effort in cultivating and transferring antigens to the DCs. Therefore it is observed that optimization in Figure 5 results in a lesser total dose. For further cost functions associated with gradients on immunotherapy optimization we refer to [13].

Melanoma is well known for its immunogenic properties usually used for immunotherapy testing. For example, one of the most cited studies on cancer immunotherapy [14] consisted in a vaccine trial of 440 patients where 96% of the patients had melanoma, although only 3.8% of the patients responded to the vaccine and the majority was from melanoma. Corresponding patients can be found suffering from lung, childhood, and kidney cancer. This could motivate the creation of mathematical models for therapy optimization on such types of cancer.

**Figure 6:**
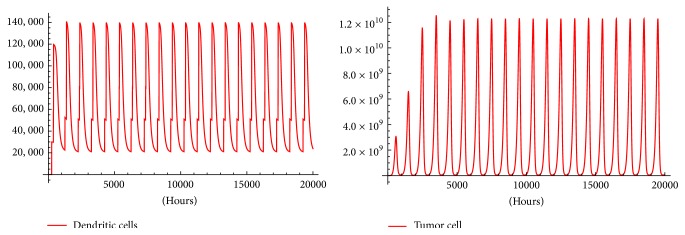
The oscillations made by the tumor with *e*_*f*_ = 0.05. In the fourth oscillation the tumor reaches as it seems to be its maximal amplitude with 1.3 × 10^10^. Then rough stable oscillations with amplitude 10^10^ occur. Both amplitudes are less than *M*_max_. Figures were generated with Algorithm 2 using inputs: *t*_*f*_ = 20,000, *m* = 20, *n* = 4, *V* = 6 × 10^5^, *x*_0_ = (6 × 10^4^, 0,0, 0,0, 0,0, 0), and a random *S*_0_ = {85,367,564,569}.

**Figure 7:**
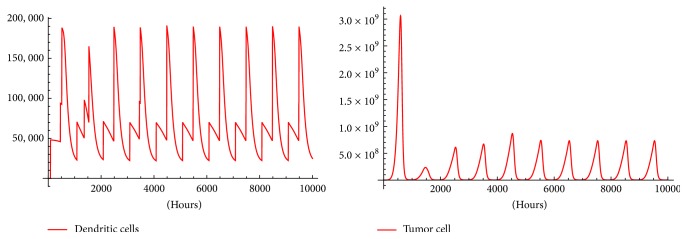
The oscillations made by the tumor with *e*_*f*_ = 0.08. In the first oscillation the tumor reaches as it seems to be its maximal amplitude with 3 × 10^9^. Then rough stable oscillations occur with amplitude 10^8^, both amplitudes less than *M*_max_. Figures were generated with Algorithm 2 using inputs: *t*_*f*_ = 10,000, *m* = 10, *n* = 4, *V* = 6 × 10^5^, *x*_0_ = (6 × 10^4^, 0,0, 0,0, 0,0, 0), and a random *S*_0_ = {85,367,564,569}.

**Figure 8:**
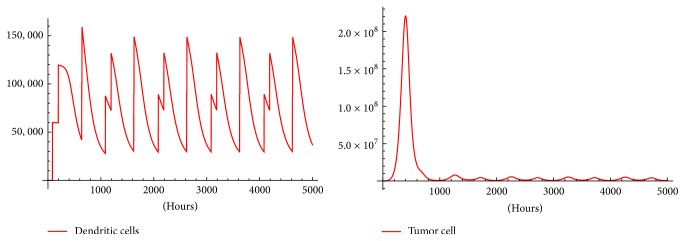
The oscillations made by the tumor with *e*_*f*_ = 0.1. In the first oscillation the tumor reaches as it seems to be its maximal amplitude with 2 × 10^8^. Then rough stable oscillations occur with amplitude 10^6^, both amplitudes less than *M*_max_. Figures were generated with Algorithm 2 using inputs: *t*_*f*_ = 5000, *m* = 5, *n* = 4, *V* = 6 × 10^5^, *x*_0_ = (6 × 10^4^, 0,0, 0,0, 0,0, 0), and a random *S*_0_ = {85,367,564,569}.

It can be observed in Figures 6, 7, and 8, a periodically pulsed immunotherapy can be used to control the tumor. This results in what seems to be periodical tumor solutions. Are these periodical solutions stable? This can be investigated creating a map *F*(*x*_*o*_) = *x*(*σ*) where *x*(*σ*) is the solution of (1)–(8) at *t* = *σ* with initial condition *x*_0_. Then, the existence of periodical solutions is equivalent to solve the equation

(13) $$\begin{array}{c} F\left(\;x_{o}\;\right)-x_{o}=0. \end{array}$$

The stability is given by the Jacobian of the map *F*(*x*_*p*_) where *x*_*p*_ is a periodic solution. These stable periodic solutions can be regarded as more robust than the unstable ones.

## 6. Conclusions

A model has been created that includes several of the obstacles that DCs face in the host environment such as immunosuppression and poor transference to the lymph nodes. The model shows an agreement with experimental data from mice inoculated with melanoma. This gives us more confidence about the optimizations outcome.

We used optimal control as a tool to rationalize the creation of immunotherapy protocols. These protocols can serve the therapist to complement their intuition and experience. Simulations show that optimized protocols outperform that proposed by the therapists in [7] regarding the total dose and final tumor size.

Although the real clinical efficacy of DC therapy is still under discussion, the results of the present work should be tested in experiments. The simulations show that an increment in the percentage of DCs that manage to arrive to the lymph nodes (*e*_*f*_) has a huge impact on the amplitude of the oscillations made by the periodic therapy. Even an increment from 5% to 10% reduces the amplitude from the order of 10^10^ to the order of 10^6^ as is shown in Figure 8. This could be achieved using better methods to deliver DC vaccines or combining DC immunotherapy with treatments that enhance the DCs migration capacity.

Can immunotherapy be a cure for cancer? That is too early to be answered. What is sure is that most of the current therapies rely on prolonging the survival of the patient and not the cure. The simulations shown in Figures 6, 7, and 8 support the idea of DCs immunotherapy as a medium to tumor control.

## Figures and Tables

**Table 1:** Immunotherapy protocol used in the experiment.

Infusion times	Week 1 (0 h)	Week 2 (168 h)	Week 3 (336 h)	Week 4 (504 h)	Week 5 (672 h)
Control mice	6 × 10^4^ Melanoma cells				Mice sacrificed
Melanoma mice	6 × 10^4^ Melanoma cells	10^6^ DCs	10^6^ DCs	10^6^ DCs	Mice sacrificed

**Table 2:** Model parameters.

Parameter	Description	Values	Units h = hours, c = cell	Reference
*aH*	Birth rate of CD4 T	10^−4^	ch^−1^	[10]
*μ*_*H*_	Death rate of CD4 T	0.005	h^−1^	[10]
*r*_*H*_	Max proliferation of CD4 T	1	c^−1^h^−1^	[10]
*K*_*H*_	Carrying capacity of CD4 T	1	c	[10]
*a*_*C*_	Birth rate of CD8 T	10^−4^	c^−1^h^−1^	[10]
*μ*_*C*_	Death rate of CD8 T	0.005	h^−1^	[10]
*r*_*C*_	Max proliferation of CD8 T	4*∗*10^−7^	c^−1^h^−1^	[10]
*K*_*C*_	Carrying capacity of CD8T	1	c	[10]
*r*_*T*_	Tumor growth rate	0.002	h^−1^	Ad hoc value
*a*_*T*_	Maximum efficiency of cytotoxic cells	0.1136	h^−1^	Ad hoc value
*τ*	DC arriving time delay at lymph nodes	232	h	Ad hoc value
*K*_*T*_	Tumor carrying capacity	10^12^	c	[6]
*μ*_*D*_	Rate death of DCs	0.009625	h^−1^	[7]
*r*_*I*_	IL-2 production by CD4 T	10^−2^	c^−1^h^−1^	[10]
*μ*_*IC*_	IL-2 uptake by CD8 T	10^−7^	c^−1^h^−1^	[10]
*μ*_*I*_	IL-2 degradation rate	10^−2^	h^−1^	[10]
*e*_*T*_	Michaelis constant	50	c^−1^	[6]
*a*_*T*_*β*__	Michaelis constant	0.69	none	[6]
*e*_*T*_*β*__	Michaelis constant	10^4^	pg	[6]
*r*_*T*_*β*__	Production rate of TGF-*β*	5.57 × 10^−6^	pg(ch)^−1^	[6]
*μ*_*β*_	Degradation rate of TGF-*β*	6.93	h^−1^	[6]
*a*_*γC*_	Release rate per single CTL	1.02*∗*10^−4^	pg · c^−1^ · ml^−1^ · h^−1^	[6]
*μ*_*γ*_	Degradation of *F*_*γ*_	0.102	h^−1^	[6]
*g*_*M*_*l*__	Receptor production	1.44	rec · c^−1^ · h^−1^	[6]
*a*_*M*_*lγ*__	Maximal effect of IFN-c	2.89	rec · c^−1^ · h^−1^	[6]
*e*_*M*_*lγ*__	Michaelis parameter	3.38*∗*10^5^	pg	[6]
*μ*_*M*_*l*__	Degradation of MI	0.0144	h^−1^	[6]
*e*_*f*_	Active DC percentage	0.05	c	[8]
